# An Exploration of How Biophilic Attributes on Campuses Might Support Student Connectedness to Nature, Others, and Self

**DOI:** 10.3389/fpsyg.2021.793175

**Published:** 2022-04-13

**Authors:** Susana Alves, Gowri Betrabet Gulwadi, Pia Nilsson

**Affiliations:** ^1^Department of Social and Developmental Psychology, Sapienza University of Rome, Rome, Italy; ^2^School of Applied Human Sciences, University of Northern Iowa, Cedar Falls, IA, United States; ^3^Independent Researcher, Lund, Sweden

**Keywords:** biophilic attributes, campus outdoors, student connectedness, student restoration, quality of life

## Abstract

University Campuses remain important settings for nurturing and supporting student health and quality of life (QoL). Research shows the health benefits of nature experiences may be facilitated by campus spaces and activities that afford connectedness. Connectedness to nature, others, and self may allow students to cope with mental fatigue, stress, and a constant need for restoration. Despite recent encouraging trends, we still lack an integrative conceptual framework to describe the mechanisms involved in achieving connectedness for making recommendations for campus design. In this conceptual review, we examine students’ connectedness in campus settings in relation to biophilic elements and attributes. We aim to understand how both direct and indirect pursuits in nature and also place-based experiences on campus foster connectedness and consequently impact students’ health and QoL. Our analysis shows that connectedness seen through the lens of Kellert’s biophilic design principles and aided by Alexander’s pattern language provides a relational and long-term perspective on recommending strategies for connecting students to nature, to others, and to themselves in campus settings.

## Introduction

Humans have a basic need to belong to a community, to connect with other people, and to become a valuable member of a group (Fiske, 2004). Drawing on Fromm (1964/1976); Wilson (1984), and Kellert and Wilson (1993) used the term, *biophilia* to describe human innate affinity and interest in forging connections with the natural world. Some forms of human experiences in environments with biophilic elements and attributes can satisfy this need to connect, belong and derive psychological benefits (Clayton, 2003; Mayer et al., 2009). Kellert offers a classification of biophilic design as comprising of two dimensions (i.e., organic or naturalistic), six elements (e.g., environmental features, natural shapes, natural processes, etc.), and 70 attributes. This matrix of dimensions, elements, and attributes illustrates the practical application of biophilic design in the built environment to enhance human functioning by offering the means for human connections with nature (Kellert et al., 2008; Kellert and Calabrese, 2015).

When biophilic attributes are available in environments, the biophilic potentiality of human-nature interactions is unleashed and activated. In other words, the latent biological tendency that humans have to connect to nature is facilitated in built environments, such as university campuses, with biophilic elements and attributes. Thus, green campuses play a key role in perceived student restoration and quality of life (Hipp et al., 2016; Gulwadi et al., 2019). This paper explores how biophilic potentiality can be identified and nurtured in campus settings through the nuanced concept of student connectedness.

There are different approaches to connectedness to nature. Evolutionary explanations based on the biophilia hypothesis emphasized in this conceptual paper focus on the affective link and deep emotional connection with different elements of nature. Other approaches define nature connectedness as: nature relatedness, which comprises of an affective, cognitive, and physical connection to nature (e.g., Nisbet et al., 2009); environmental identity (e.g., Clayton, 2003), inclusion in nature (Schultz, 2002), ecological identity (Walton and Jones, 2018), and a sense of oneness with nature (Lengieza and Swim, 2021) among others. There is evidence that spending time in nature, engaging with nature directly and indirectly in a variety of forms (*via* walking in nature, nature-based tourism, living closer to nature, and immersive experiences provided by virtual reality), and a strong sense of nature connectedness positively affects well-being (Richardson et al., 2021).

As little as 10–20 min of time spent sitting or walking in nature shows a beneficial effect on college-aged adults’ mental health (Colding and Barthel, 2017) when compared with similar time spent outdoors in urbanized environments. Even though green campuses are aimed at student recruitment, the health-related benefits accrued through interactions with and within campus landscapes, especially in a post-pandemic world, are still poorly understood. Students are often unaware of the ecological importance of green spaces on their campuses (Speake et al., 2013) even though their choice of university critically depends upon perceptions and evaluation of outdoor spaces (Groen and White, 2003). Campuses offer unparalleled place-based learning which can be complemented by the potential for deep connections with, and use of their green spaces. To develop biophilic design on campuses, we must first conceptualize the mechanisms with which students perceive, experience, and connect emotionally to campus nature before we can identify what types of *accessible and sustainable “doses” of nature* elicit a positive impact on *mental health* (Meredith et al., 2020). We define connectedness as passive and active engagement with natural elements and attributes on campus and propose that these mechanisms facilitate student connectedness at multiple levels.

Connectedness to nature is significantly linked to both hedonic and eudaimonic well-being (Capaldi et al., 2015; Whitten et al., 2018). The hedonic approach defines well-being in terms of pleasure attainment and pain avoidance (e.g., focus on happiness) whereas the eudaimonic view focuses on individuals’ functioning, meaning, and self-realization. Studies suggest the well-functioning aspects of well-being (i.e., eudaimonic well-being) are more strongly associated with nature connectedness than those related to hedonic well-being (e.g., Howell et al., 2011; Capaldi et al., 2014). This is because eudaimonic and hedonic well-being tend to be associated with different motives, behaviors and experiences. Eudaimonic behaviors may lead to experiences of meaning making, elevating experiences and sense of connection with a greater whole (Henderson et al., 2013).

Positive exposure to nature at a local level within educational settings may eventually generate concern for abstract or global environmental issues, and students may be more likely to bring that experience into their later professional and private relationship to nature. Interconnectedness and dependence on nature could propel environmental conservation action (Restall and Conrad, 2015). Some people also identify themselves as part of nature and equal to other life forms (Clayton, 2003) which may also serve as motives for concern for environmental problems. Both connectedness and integration promote student satisfaction, academic success, and retention (Jorgenson et al., 2018) and can be fostered in higher education (Lankenau, 2018).

The recent COVID-19 pandemic disrupted human communities, social networks, and overall quality of life, creating a great need for restoration, self-regulation, and social contact. Individual-level connections with the soft characteristics of nature such as vegetation, pets or wild birds, might to some extent compensate for reduced post-pandemic connectedness among people returning to campus. During lockdown times, restorative needs can be met through direct (i.e., gardening) and indirect (i.e., view from window) engagement in nature (Soga et al., 2021; Theodorou et al., 2021). Other pandemics will happen in the future (IPBES, 2020; UNESCO, 2021; report on biodiversity and pandemics) pointing to a continued need to counter isolation and safeguard well-being through biophilic environments. The innate human need to connect to life, nature, and life-like processes (i.e., biophilia hypothesis proposed by Kellert and Wilson, 1993) and its repression during pandemic-induced confinement situations highlights two key findings: (1) that nature connectedness may be a key mediator in the relationship between nature engagement, QoL, and psychological well-being and; (2) a pressing need to develop strategies to translate what is innately healing to the design of campus settings.

Connectedness to nature is key in mediating the association between exposure to greenery and students’ well-being (Van den Bogerd et al., 2018), is positively related to self-reported and actual pro-environmental behavior (Mackay and Schmitt, 2019; Whitburn et al., 2020) and enhances a sense of belonging which is linked to student retention and success. Nature-connected people also empathize with non-human lives, show a low social dominance orientation, and are more likely to connect with other human beings and offer support to marginalized groups (Ng and Leung, 2021). Consequently, the significance of feeling connected with nature is important in enhancing psychological growth and involves more than just spending time in nature (Pritchard et al., 2020).

While the biophilic approach has been applied to understand how urban settings can help people with a ‘daily dose of nature’ (Beatley, 2010), it is challenging to apply biophilic principles to actual campus design and management (Abdelaal, 2019). A translational approach requires an adaptive pattern language (i.e., living patterns; Alexander et al., 1977; Kahn et al., 2010; Salingaros, 2017) to guide spatial design and planning. We propose a relational view of nature to acknowledge the multi-faceted impact of physical and spatial features of green campuses on health and well-being (Chan et al., 2018). Additionally, a lens of student connectedness helps identify mechanisms that better translate abstract biophilic attributes to concrete design application. Student connectedness on green campuses can be explored at three levels—connectedness to nature, to other people, and to one’s self—to help build an explanatory path between campus features, good health, and QoL.

This conceptual paper provides a knowledge basis to frame, understand, and promote biophilic design and nature-based interventions on campuses by:

• Proposing a conceptual framework to understand student connectedness in campus settings and its relationship to student health and QoL, and
• Illustrating its usefulness by discussing specific biophilic design patterns and how they can facilitate greater student connectedness with nature, others, and one’s self.

Campuses are social–ecological systems to the extent that they can offer a context and new opportunities for people to connect to nature. Addressing the dynamic and diverse physical and functional relationship between the campus and the use of its spaces by students, educators, and staff through this framework can help university stakeholders to first understand the campus potential and then improve the design decisions they make.


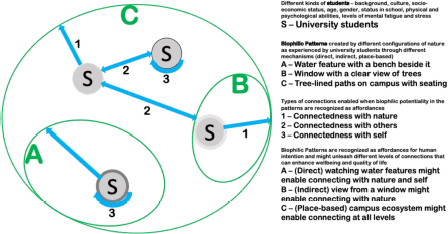


*In this conceptual framework, university students of different backgrounds and with different restorative needs (S) encounter natural elements and attributes configured according to biophilic patterns on campuses. During these everyday encounters, students experience these biophilic patterns (e.g., A, B, C) through direct (e.g., sitting on a bench and viewing a water feature), indirect (e.g., gazing at a view outside a classroom window), or place-based (e.g., walking through campus) experiences. When biophilic patterns are perceived as affordances, that is, having the potential for health-promoting activities and interactions, students experience either connectedness with nature (1), with others (2), and/or with themselves (3). These everyday connections can further enhance their sense of well-being and quality of life. This everyday, dynamic process can also vary according to the needs of the student, recognition of affordances offered by the biophilic campus patterns, the mechanism of interaction, and the type of connectedness achieved.*

## A Relational View of Quality of Life

Connectedness to nature is closely linked to people’s QoL (Olivos and Clayton, 2017). The World Health Organization (WHO) defines QoL as an individual’s perception of their position in life in the context of the culture and value systems in which they live and in relation to their goals, expectations, standards, and concerns. It is a broad ranging concept affected in a complex way by the person’s physical health, psychological state, level of independence, social relationships, personal beliefs and their relationship to salient features of the environment (WHO, 1997).

QoL varies with the cultural context and value systems in which individuals live, and in relation to their goals, expectations, standards, and concerns. Students’ use of campus green spaces is related to perceptions of QoL (McFarland et al., 2008). A relational concept of QoL and well-being emphasizes a multidimensional, needs-based approach that involves the satisfaction of both material and less tangible needs, and explores students’ connection with specific qualities and attributes of the campus environment.

In a relational view of QoL, individuals, groups, social and physical settings are seen in a state of constant change. Qualities often change with people’s experiences of place and also with the consequences of their direct and indirect contact with it. Alexander (1979, p. 19) proposed that “there is a central quality which is the root criterion of life and spirit in a man (sic), a town, a building, or a wilderness, it is objective and precise, but it cannot be named” and embodies a “freedom from inner contradictions.” He offers different enduring spatial patterns that translate how this quality can be achieved (Alexander et al., 1977). Recasting Alexander’s notion of “quality without a name” with that of “quality with many names” helps understand and situate students’ connectedness and varied experiences in campus settings. For campus design interventions, we limit this discussion to the study of nature and outdoor spaces and specify the “qualities” by considering them as “affordances” (Gibson, 1979) or potentialities of action that are provided by campus nature both in tangible and more experiential terms (Alves, 2018). Affordances are relational and processual in character (Heras-Escribano and De Pinedo-García, 2018) and help designers and researchers in translation. Our premise is that a sense of connection (with nature, others, and one’s self) will enable people to be the best version of themselves and reach their full potential (Kaplan and Kaplan, 2008). Campus affordances must therefore support students to reach their full potential. By tapping into their range of experiences and need for connectedness, we can begin to qualify QoL with different names in tangible ways.

## Connectedness to Nature in Campus Settings

Connectedness to nature is a relational process that refers to people’s identification with the natural world and the relationships they establish with it (Restall and Conrad, 2015). Connectedness viewed as a “transaction” (Dewey and Bentley, 1989/1949) explicitly links perception and action similar to Gibson’s notion of affordances (Gibson, 1979). Recognizing affordances depends on people’s characteristics, cultural context, and experiences. Educational settings and campuses are social-ecological systems in which people’s *transactions* with green spaces can be analyzed in terms of connectedness (Heymans et al., 2019).

Kellert’s (2005) biophilic design principles include three *strategies for connectedness*: direct contact with nature, indirect/symbolic contact with nature, and experiences of space and place—that relate humans and the built environment or landscape within a specific cultural context, and can *activate people’s biophilia*. A framework of six biophilic design elements (environmental features; natural shape and forms; natural patterns and processes; light and space; place-based relationships and evolved human-nature relationships) are embedded in more than 70 biophilic design attributes (Kellert, 2012) and have been further revised and simplified (see Kellert and Calabrese, 2015). This framework is supported by study findings in different disciplines (Browning et al., 2014), but so far has not been related comprehensively to campus settings.

Biophilic design is not merely about introducing trees and vegetation (e.g., green roofs, green walls, water sensitive urban design) into built settings—it consists of eliciting biophilic responses such as restorative moments (Gifford and McGunn, 2012; Gillis and Gatersleben, 2015) as part of the overall built environment experience. We propose that direct, indirect, and place-based experiences (the basic tenets of biophilic design) on campuses can lead to connectedness—to the place/campus, to other people, and to one’s self. Biophilic design attributes can also be considered as “histories of connection” which encompass individuals, the environment, and others (Ingold, 2011). Thereby, activities in natural environments involve a “loop of information” where tasks, tangible, and intangible dimensions intermingle. Activities are not undertaken against the backdrop of the built environment, they are imbued in the environment itself—“the environment is a world that continually unfolds in relation to the beings that make a living” (Ingold, 2011, p. 30).

Perceived environmental affordances on campus settings are actualized by people’s embodied acts, such as walking, looking, smelling, hearing, etc. People create sensorial links to the immediate environment through “histories of connections” that sustain a high QoL. Through the lens of connectedness, student’s experiences and actions in campus settings are categorized as:

(1) Connectedness to nature: how students relate to specific environmental attributes on campus; that is, how they move on campus, and what they see, hear, smell, and touch.

(2) Connectedness to others: how students connect with others and use campus spaces for gathering through shared activities.

(3) Connectedness to themselves (in nature): how students rest, find respite, inward focus, and achieve restoration and self-regulation while relating to nature in both direct and indirect ways.

Using these three levels, we will review biophilic patterns, attributes, and their qualities associated with students’ experience and use of diverse configurations of green and open spaces in campus settings.

### A Framework for Biophilic Design Through Connectedness

We propose that biophilic design attributes are made up of properties that represent a “condensed story” involving people, nature, and others (Ingold, 2011). To describe the properties of materials is “to tell the stories of what happens to them as they flow, mix, and mutate” (Ingold, 2011, p. 30). The three overarching types of biophilic connections: connectedness to nature, others, and self; the three kinds of mechanisms: direct, indirect, and place-based, and the types of benefits students gain from these connections: cognitive, physiological, and psychological help structure a biophilic design pattern language.

A pattern language is considered a language of archetypes (Alexander et al., 1977). The design patterns are intended to enable “archetypal natural elements and configurations” to be expressed in landscape and urban design. As this conceptual paper illustrates, there is enough evidence to support the need for a pattern language in campus settings. A language of patterns makes it possible to describe the overall problem of student connectedness to nature by considering problem, solution, research, design implications, and consequences to human health. *Patterns are hypotheses* that can be tested in empirical research but are also instructions given to designers as an overall framework for working out a design solution. Due to its openness, a pattern language is a *valuable catalyst* that can be tested and applied to a variety of contexts such as campus settings. Acquired *via* observations, empirical research, and design practice, a pattern language helps us articulate and activate the human connectedness to nature. Next, we review empirical evidence to link measures of QoL and health to these three kinds of connection (nature, others, and self) by aligning them with relevant biophilic design patterns.

#### Connectedness to Nature

Connectedness to nature has been studied as a personal disposition relevant for environmental health, human health, and intergroup attitudes and behavior (Ng and Leung, 2021) that includes direct and indirect contact with nature and natural processes. We discuss four related biophilic design patterns: visual and non-visual connection to nature, presence of water, and connection with natural systems.

##### Visual Connection to Nature

Visual connection to nature defined as visual access to elements of nature, living systems and natural processes (Browning et al., 2014) can be restorative (Kaplan et al., 1998), especially if there are large windows to the outdoor landscape allowing expansive views (Appleton, 1975/1996). People prefer natural views over built views and derive restoration from mental fatigue and stress after engaging with nature (Kaplan, 1995; Hartig, 2007).

Students perform better on standardized exams, and finish high school at higher rates when their schools offer views of landscapes with greater quantities of trees and shrubs from the cafeteria and classroom windows (Matsuoka, 2010). A majority (97%) of students preferred a natural view from their windows and also appreciated green views even if the duration of experiencing the natural view was brief—between 5 and 10 minutes (Lau and Yang, 2009). Actual greenness at three different spatial levels—overall campus, central campus, and near academic buildings—was correlated with student perceived greenness in Turkey, whereas in the U.S.A., it was correlated only with central campus greenness (Gulwadi et al., 2019). Visibility and access to views from windows of academic buildings may have played a role in differences of perceived greenness. The type of campus trees viewed by students in immersive scenes restored them differently or more deeply from a stress-inducing activity (Guo et al., 2020). Physiological (R-R heart rate interval, alpha and beta waves of electrical brain wave activities) and psychological (state anxiety and perceived restorativeness) measures showed there were positive benefits of watching each tree, but viewing certain species like the Gingko tree conveyed the most restorative benefits. Thus, actual or virtual visual connection with multiple green areas with various green elements allow for different affordances to be actualized, as they enlarge the range of activities students can pursue to better accommodate their diverse restorative needs (Gulwadi et al., 2019).

##### Non-visual Connection to Nature

Ryan et al. (2014) characterizes non-visual connection to nature by auditory, haptic, olfactory, or gustatory stimuli that engender a positive reference to nature. Grahn and Stigsdotter (2010) provide a set of *perceived sensory dimensions* that emerge from embodied relations with the environment—affiliating with nature through these qualities results in restoration from stress. Bird songs and wind promoted restoration over hearing no sound in a park experience (Abbott et al., 2016) and nature sounds had a stronger stress recovery effect through skin conductance levels in a study of adults exposed to nature or noisy environment sounds after a stressful calculation task (Alvarsson et al., 2010).

In a beach setting, walking barefoot mediated nature connectedness and psychological restoration (Rickard and White, 2021). Other immersive experiences, such as walking in a forest were associated with significant reductions in physiological measures (i.e., systolic blood pressure) and with positive feelings (Park et al., 2010). Also, Volatile Organic Compounds (VOCs) (e.g., limonene, alpha-and beta-pinene, beta-myrcene, and campene) emitted by different plant species are beneficial to human health (Antonelli et al., 2021).

Young adults recovering from attentional tasks through either a nature walk, in a room with tree views, or a room with no views showed reduced ambulatory blood pressure, decreased anger, and increased positive affect (Hartig et al., 2003) after the nature walk. Psychological measures such as perceptions of mental health and tranquility improved after non-visual sensory interactions with non-threatening nature (Grahn and Stigsdotter, 2003).

Students report higher QoL when they perceive their campus to have higher levels of “greenness” (Hipp et al., 2016). For students with stressful lifestyles, perhaps just perceiving access to nearby nature can influence their well-being. Objective indicators of greenness, such as density, proximity, type, size, and quantity may influence students’ satisfaction, health, and performance (Gulwadi et al., 2019). Interactions with campus nature may exist (i.e., affordances are available) but might not be actualized, discovered or explored further. Although visual connection to nature predominates, nature experiences are multi-sensory, and perceived sensory dimensions are also key to students’ experience.

##### Presence of Water

Water elicits high preference ratings and positive emotional responses (Ulrich, 1983). Natural environments and built scenes containing water are associated with higher preferences, greater positive affect, and higher perceived restorativeness than those without water (Heerwagen and Orians, 1993; White et al., 2010; Windhager et al., 2011).

A majority of students identified natural settings with water features as their refuge when experiencing high levels of stress (Francis and Marcus, 1991). A study examining the restorative potential of real and simulated landscapes for students, found that landscapes having a lot of water were high in restorative potential (Felsten, 2009). A majority of Hong Kong University students prefer an outdoor campus area where water is present (Lau and Yang, 2009). Students associate waterfront spaces with optimal perceived attention restoration effect, followed by vegetation spaces, courtyard spaces and square spaces (Lu and Fu, 2019). The integration of water elements in university design is scarce (Peters and D’Penna, 2020) but studies have revealed that images of nature depicting water are restorative and images of study areas with a water feature are preferred (Hami and Abdi, 2019). Also, when combined with diversified vegetation and various levels of privacy, water scenes provide positive distraction.

##### Complexity and Order

Complexity and order refer to how much information is present in a space and how it is organized. The level of complexity in campus settings refers to the presence of sensory information that is configured in a coherent way, with a coherent spatial hierarchy and how specific configurations lead to positive or negative outcomes (Klinger and Salingaros, 2000; Salingaros, 2006). It is also related to the patterns of interaction between diverse system elements, at different levels and times, and the emergence of new interactions (Thompson et al., 2016). In sum, complexity and order is more than just analyzing individual elements in isolation and looking for the presence and amount of visual information.

In a physical environment, order is associated with spatial arrangement, and the extent to which different elements are coherent, legible, and/or clear (Nasar, 1997). An ordered environment lacks informational stress and supports basic human needs for understanding and exploration (Kaplan et al., 1998). Natural scenes that communicate a sense of orderliness (e.g., parklike areas with smooth ground texture) make it possible to acquire and make sense of information in the environment (Kaplan, 1985).

Natural environments are preferred over built ones because of their level of naturalness and also due to their order and structural morphological properties. Low preference ratings for built settings are associated with a lack of organized complexity and non-biophilic “unnatural architectural styles” (Salingaros, 2019). Organized visual complexity corresponds to the concept of “wholeness” or coherent structure (e.g., Alexander in *The Nature of Order*) and is important in terms of preference and consequent positive experience. A strong correlation between “enriched environments” measured by the coverage of forest, and amygdala integrity revealed that forests have salutogenic effects for people living near them in Berlin (Kühn et al., 2017). A weak correlation between amygdala integrity and urban green areas is perhaps because these areas contain a low degree of organized complexity (i.e., lawn, or isolated bushes and trees) when compared to the forest.

The organization and complexity of the environment is also related to a sense of coherence. For instance, a walk in a well-maintained urban park with giant trees and natural views is seen as “a more coherent” and better environment for regeneration and positive mood than a walk on an urban street (Aziz et al., 2021). Students prefer scenes with high levels of coherence and legibility when compared to scenes with low levels of coherence and legibility (Hami and Abdi, 2019). An interesting and legible environment may help students feel compatible with their surrounding campus environment, and is more likely to enhance QoL (Gulwadi et al., 2019). A good balance is needed between an interesting information rich environment that is restorative, and one with too much information that may be perceived as stressful and confusing (Kaplan and Kaplan, 1989).

Natural geometries, as well as shapes and forms, have defining qualities that we perceive as approachable or avoidant. Visual patterns that mimic or refer to biological and natural patterns have a geometry that draws interest and connects us to nature. Biophilic design proposes an innate response to the specific geometry of natural forms, detail, hierarchical subdivisions, and color, among others (Salingaros and Masden, 2008). In campus settings, green and built spaces have intrinsic qualities that enable students to connect to nature and gain benefits. Fractal qualities (i.e., ordered details arranged in a nested scaling hierarchy) contribute positively to well-being (Hagerhall et al., 2004; Taylor, 2006).

On campuses, there is often a sharp contrast between building forms and the surrounding natural environment. Plants within a building, or in a building’s garden or courtyard may soften this contrast but designing buildings using a complex built geometry in synchrony with natural forms can also enable us to connect fractally (Salingaros and Masden, 2008). Salingaros (2020) proposes using living forms and geometric characteristics (inspired by vernacular architecture) in his toolbox for building and repairing a campus, as irrelevant non-contextual forms may not afford the desired level of connectedness. Biophilic design patterns on campuses thus act as geometrical connective rules that may affect our neurophysiology in a direct way (Alexander, 2002–2005).

##### Connection With Natural Systems

Campuses can reconnect people to the biosphere by fostering a transition toward sustainable development and social–ecological system sustainability (Colding and Barthel, 2017). Biophilic patterns allow connections with plant and animal life in a way that supports ecosystems and native plant species (Salingaros and Masden, 2008). University campuses are considered small cities since they are directly connected to the larger environment (i.e., urban, agricultural/natural or a combination of these) in which they are situated (Alshuwaikhat and Abubakar, 2008; Finlay and Massey, 2012). A “Biophilic City” approach to campus settings (Ratajczyk et al., 2017) can enable people’s actions within this system to greatly contribute and enhance ecosystem services and bring positive social and ecological outcomes (Tidball and Stedman, 2013).

Nonetheless, the recognition that campuses can afford connectedness between diverse systems at diverse scales, habitat preservation, and ecosystem integrity has received low importance in sustainability planning in university campuses (Orenstein et al., 2019). Finlay and Massey (2012) have shown campus areas may represent protected *niches for sustainability* by integrating ecological considerations into a campus spatial planning. Visual and non-visual interactions with trees, plants, small animals, birds, and water bodies represent proximal everyday resources for students to engage with on campus. Research exploring how interactions with animals in classroom settings impacts learning motivation, engagement, self-regulation, and human social interaction found that activities with companion animals can stimulate curiosity and learning while also providing a source of emotional support (Gee et al., 2017). On campuses, universities offer pet therapy programs for students (i.e., The University of Connecticut’s Homer Babbidge Library) and provide therapy dogs to support the physical and emotional well-being of students during the stress-filled week of finals with positive outcomes (Reynolds and Rabschutz, 2011). Additionally, bringing in animals into campus dormitories for emotional support is becoming increasingly acceptable.

Although connection to natural systems is multisensory, the vast amount of research on the health benefits of green spaces primarily deals with visual perception. Appleton (1984) connects people’s multisensory experience with the ideas of prospect and refuge. He reminds us that the lack of boundaries between spaces expands sensory awareness that can be evoked in campus settings with multiple view corridors and the opening up of interior and exterior vistas. The contemplation of the horizon is greatly impeded in campus settings but it has an important role in providing soft fascination, mystery, and changing one’s attention.

Biophilic connections to natural systems are also present in cycles of growth and decay, such as age, change, and the patina of time. Many aspects of decay, such as decaying animals, dirty water, and dark places may lead to dislike, anxiety, fear, and avoidance and can elicit ‘biophobia’ (Kellert et al., 2008). However, some elements of decay that signal the passage of time may in fact lead to positive responses in campus settings. For example, decaying trees may be disliked (Tyrväinen et al., 2005) but they are rich resources for a variety of plants and animals (Kellert et al., 2008). The seasonal rhythms of leaves falling in autumn are a sign of decay and regeneration that is widely accepted as an annual event to enjoy.

On campuses, community-campus partnerships in gardening activities involving students, faculty, older adults in community residential care facilities, and daycare children helped foster healthy relations with, self, others, and the environment (Jakubec et al., 2021). Campus-community garden initiatives can thus be a transformative pedagogy serving the purposes of both fostering interpersonal relations and ecological goals. Green campuses can provide diverse ecosystem services by integrating greenery (e.g., planting trees around sidewalks, between buildings, and in unusable areas) to create green corridors and roofs. Open spaces in campuses serve as a micro-ecosystem for plants and wildlife and also for people. Gardens and green roofs provide space for growing food, which can be used to teach students about food sources, agricultural practices, and nutrition (Lau et al., 2014).

Natural ecosystems are usually well-connected systems (i.e. with flow of materials and organisms across boundaries). Thus, the discussion of connection to natural systems also involves the issue of how a university and its campus spaces and programs can best combine its traditional teaching and research role with new roles in regional economic development. For example, green campus solutions in open spaces can successfully reduce ecological footprints (Genta et al., 2019).

Connections to natural systems on campuses have been studied in relation to micrometeorological conditions (Lin et al., 2013). Research has shown that levels of physical activity vary with seasonal changes with wet and cold seasons acting as barriers to participation in physical activity (Tucker and Gilliland, 2007). Also, with change in seasons and drop in temperature, the number of people who often use outdoor spaces is greatly reduced (Shooshtarian et al., 2018). Campus greening is an effective method for reducing ambient air temperatures and for providing diversity of activities in different seasons (Srivanit and Hokao, 2013).

Enhancing connection to natural systems on campuses may require a triple helix model linking teaching, research, and development (e.g. Etzkowitz and Leydesdorff, 2000) to establish cooperation between regional governments and universities. There is a need to recast the role of university campus landscapes within a learning ecosystem through educational and leisure outdoor activities (Scholl and Gulwadi, 2018). Taking part in outdoor education programs has positive outcomes on students’ psycho-physical well-being, connectedness to nature, and pro-social behavior (Pirchio et al., 2021).

The ecology of health requires that we approach biodiversity at different scales. At the micro scale, humans have 100 trillion microorganisms living inside their bodies (Mayer et al., 2014). Environments that promote diverse microbial communities often correlate with those with greater diversity of macro-flora and fauna (Flies et al., 2017; Robinson et al., 2018). Neuroscience research demonstrates that exposure to microbial species found in natural environments positively influences human immune responses and impacts well-being. The body’s natural array of microbes may alter brain functions in beneficial ways to decrease anxiety, depression and other mood disorders (Schmidt, 2015; Mayer et al., 2014). The finding that gut microbiota have therapeutic potential as they regulate stress, anxiety, and cognition points to the need to increase diversity of green spaces on campuses to better understand the link between microbes and behavior (i.e., gut-microbiome-brain interactions) and to promote environmental education focusing on the macro and micro benefits of biodiversity for human health.

Expanding people’s connectedness to other beings and other species (Cooke et al., 2020) is a way to acknowledge and respect non-human agency. When individuals extend their self-definitions to include the more-than-human-world, they tend to act in an eco-friendly way towards the environment (Clayton, 2003), expand their sense of self, and give greater value to non-human species (Gosling and Williams, 2010). Therefore, connectedness to natural systems can be seen as a way to promote QoL in campus settings.

#### Connectedness to Others

Because the physical and non-physical campus dimensions form a community, understanding which spatial configurations might promote community building and a sense of attachment to others and to places is important. Green spaces that provide opportunities for students to engage with diverse peers in ongoing interaction are more likely to enhance connectedness. Salingaros (2020) proposes that a campus is mainly a pedestrian environment with multiple internal and external links. Next, we discuss the following biophilic design elements and patterns: spatial variability (i.e., presence of sociopetal spaces) and place-based relationships that can foster connectedness to others.

Spatial configurations of the campus setting enable or hinder social interaction and shape connectedness to others to the extent in which they have affordances for sociability. Sociopetal (or inward facing) space encourages conversations whereas sociofugal (outward facing) space does not (Sommer, 1967). Sociopetal campus spaces are user-oriented areas with amenities that provide opportunities for eye contact, have distances that permit conversation between people, and also opportunities to observe others. Thus, connectedness to people can be understood through the interconnections between material and non-material aspects within campus green spaces (Speake et al., 2013). Examining fixed-feature elements (e.g., windows, walls, ceilings, floors) and semi fixed-feature elements (e.g., furniture, street furniture, and also humans) help define and specify environmental attributes affording social behavior.

On campuses, sociopetal spatial arrangements in green spaces may enhance connectedness to others by enabling effective breaks on campus which in turn may enhance opportunities to bring people together (Osmond, 1959; Gehl, 1987). Natural scenery and a relaxing atmosphere in sociopetal open spaces encourage meetings and conversations, while providing fresh air for stressed people. Supportive campus settings have intimate-scaled spaces, informal spaces, and welcoming spaces for students to forge relationships with others, expand their social network and feel connected to a place. Gehl (1987) describes environmental conditions that can support social interaction. Boundaries, edges, spaces for containment, and issues of accessibility (i.e., pedestrian routes and their organization) and visibility are important spatial configuration elements to afford social interaction. For example, at the edge, one is less exposed than if one is in the middle of a space thus creating a quality desirable for stationary activities. Boundaries and edges of spatial configurations that demonstrate refuge/prospect facilitate social interaction as it is easier to watch and react to others. With one’s back protected, a sense of refuge is afforded while not having a blocked visual field gives prospect for exploration. Spaces for containment allow one to reduce and control one’s personal territory providing affordances for intimate encounters. They also allow people to linger and to observe others without being necessarily observed.

Low interaction spaces are characterized by the absence of intimate scale features, such as adjacent spaces for meeting and relaxing. Lack of a sequence of open spaces to connect a variety of places and integrate different areas of a campus into a holistic system leads to *sociofugal spatial arrangements* that hinder social interaction by obstructing eye contact between people and making it more difficult to establish person-to-person verbal interaction and opportunities to watch others.

The adaptive capacity to cope with stress and uncertain situations—resilience—can be bolstered when people feel connected to places and others (Faulkner et al., 2018). Students are expected to excel in academic education and focus on intellectual activities while simultaneously dealing with everyday stressors (Ribeiro et al., 2018). Students’ capacities to be resilient, such as having a strong will, not giving up, and maintaining an open mind (Mueller, 2021) demands a broader relational view (i.e., transactional) of a campus environment to identify key capacity-building affordances for resilience. Resilience on campuses may be promoted through *place-based relationships and evolved human-nature relationships* (Kellert et al., 2008), such as place attachment, knowledge and learning, and community cohesion. Connection to the ecology of the campus is one way to promote place attachment and avoid placelessness.

Students face disruption associated with relocation and transition from home to the university setting, resulting in stress and altered social networks. Inability to adapt to unfamiliar campus environments makes university students more susceptible to the occurrence of psychological disorders (Xu et al., 2015). Attachment to nature can give students a “secure base” making it easier for them to get close to others. Spending time in outdoor sociopetal spaces may be important to create social networks but may not suffice. Campus settings can fulfill specific psychological needs for place attachment when students can exercise “appropriation” (i.e., making something one’s own through using spaces and objects)—a mechanism by which attachment develops (Rioux et al., 2017). However, campus settings with predominantly sociofugal spaces might impede place attachment when students are separated from their natural surroundings during long teaching and learning hours indoors, in classrooms.

Using campus surroundings in outdoor education allows for experiential learning where students use all their senses to forge relationships with people and natural resources (Mannion and Lynch, 2016). When education is rooted in outdoor campus settings, students have a chance to transform *spaces into places they are attached to*. Non-places as defined by Augé (2008), are disconnected spaces lacking meaningful relations with other spaces and/or concern with intimacy. These “junkspaces” as coined by Koolhaas (2002), abound in universities. They are physically manifested in sterile, air-conditioned spaces of courts, lounges, and conference rooms, among others, and pose fundamental challenges for enhancing place attachment and sense of connectedness because they *lack affordances for connectedness*.

Affordances for connectedness can be found in non-sterile in-between spaces that allow both passive and active pursuits in nature (Gehl, 1987). Gardening activities may be done in such in-between spaces to allow connectedness to place, others, and self. Gardening has been linked to social capital and sense of cohesion in the community, a venue for social activities and a setting for interpersonal interaction. Community gardens, as part of urban green spaces, offer meaningful social interaction opportunities and enhance social cohesion (Veen et al., 2016).

Nature-related activities enhance sense of cohesion, social support, and sense of belonging (Maas et al., 2009). Collectively, biophilic elements and exposure to green spaces enhance perceptions of belongingness (Van den Bogerd et al., 2018). Nature-based stewardship and leisure activities in green campus settings may strengthen students’ sense of place and desire to give back to the university and larger community. Wanting to help others or contribute to the common good is a good way to develop connectedness (Krasny and Delia, 2015) which can be further reinforced by a campus climate that is more supportive of diversity (i.e., with respect to race and ethnicity).

In sum, designing campus spaces that connect people to each other is helpful for achieving sustainability goals. *Designing affordances for connectedness* means providing sociopetal spaces to foster social contact, place attachment, and further experiences such as resilience and social cohesion.

#### Connectedness to One’s Self

Connectedness to self refers to people’s perceptions and preferences for open and green spaces by taking into account their own needs, values, meanings, attachment, and need for self-regulation. Students are under pressure for directed attention which makes them prone to mental fatigue. A restorative campus that provides a sense of being away, extent, fascination, and compatibility offers opportunities for students to change the focus of their attention in order to regulate internal processes, such as ruminative thoughts (Kaplan and Kaplan, 1989). A self that is grounded in attachment to nature is more resilient and better adapted (Bowlby, 1969/1982). Natural places can serve as “attachment figures” similar to the infant-mother/caregiver attachment (Scannell and Gifford, 2014). Places of attachment (such as green campuses) are related to connectedness to self through the fulfillment of the *human need for proximity*, having a *safe haven* and a *secure base* which are key to psychological well-being (Bowlby, 1969/1982). In campuses, attachment to favorite natural spaces offer a safe haven for the reorganization of the self in stressful situations (Korpela et al., 2002). Individuals with positive views of self and others often have a secure attachment (Brennan et al., 1998).

Engagement in nature-related activities may greatly impact one’s self-identity (Olivos and Clayton, 2017). A campus setting that affords preferred activities has the potential of enhancing positive self-identity. For example, Foellmer et al. (2021) showed that most students pointed to the *Hofgarten* (a publicly and freely accessible academic green space adjacent to the main building at the University of Bonn, Germany) as important for identity-creation on their campus. Activities, such as campus reforestation have been associated with positive emotions such as a sense of pride and competence that emanate from hands-on stewardship in nature (Koester et al., 2006).

Opportunities for *experiencing prospect* (i.e., spaces that offer observation and a chance to survey the environment) and *refuge* (i.e., spaces that provide shelter and places to hide) support connectedness to self. This evolutionary ability to hide and seek refuge is demonstrated when teenagers and adults seek refuge in green environments for perceived restoration and reduced stress (Lückmann et al., 2013; Birch et al., 2020). When feeling stressed and sad, teenagers prefer places surrounded by plants rather than by people (Lückmann et al., 2013). Prospect afforded through path availability and accessibility has an impact on mood in a simulated forest hike—with no paths resulting in reported low levels of pleasure (Staats et al., 1997). Both prospect and refuge lead to restoration from stress and help overcome ego-depletion (Beute and De Kort, 2014) and foster self-regulation (either seeking others or engaging in contemplation/reflection). Moreover, refuge is a significant feature in restorative environments for stressed individuals (Grahn and Stigsdotter, 2010). A green area that has well-cut grass and football fields on grass may reduce teenagers’ stress and promote their mental health (Akpinar, 2021). In fact, students prefer a landscape rich in prospect for spending free time (Hami and Abdi, 2019).

Loose flexible spaces may help students shape the environment to suit their different needs and appropriate space (Franck and Stevens, 2006) by affording transformability/re-purpose and multi-use. Appropriation is linked to a range of psychological processes, such as creating, reflecting, being spontaneous, imagining, and sharing—all involving self-expression. Self-expression is best afforded in “loose and leftover spaces” (Stevens, 2007), especially in green/open spaces that allow temporary activities geared to acquiring meaning and place attachment. Porous edges (i.e., one can see and move easily between spaces), loose (i.e., being open to appropriation), and leftover spaces (i.e., with no assigned function) in campus settings facilitate a sense of compatibility, self-regulation, and play. Even though playfulness is usually associated with children’s behavior, it is also very important to adults’ health.

Natural settings are preferred places for self- and emotion regulation (Korpela et al., 2001). Students prefer open spaces with natural settings to ameliorate their moods when they are stressed, upset, depressed, angry or confused (Lau and Yang, 2009). The process of taking a walk in nature (as opposed to the outcome of walking) helped students feel rejuvenated, “tuned into nature” and more present during the activity (Shrestha et al., 2021). The natural environment changed their mood in a positive direction and helped them focus, prioritize and solve problems while walking. Walking outdoors, such as taking a 30-min walk in an urban park is also important to reduce ruminative thinking whereas a city walk may not have the same beneficial effect (Lopes et al., 2020). Ruminative thinking refers to frequent and repetitive self-centered analyses concerning negative self-descriptive patterns of thought—one of the most maladaptive cognitive emotion regulation strategies of mental illness. It leads to elevated inflammation and cortisol levels, impaired problem solving, and can predict substance abuse, eating disorders, and self-harm (Lopes et al., 2020).

Connection to one’s self may be encouraged through diverse passive and active pursuits, such as stretching, breathing, walking, meditating, and exercising. A study with college students in Japan showed that walking and sitting in a forest and watching the forest landscape led to a reduction of prefrontal hemoglobin concentration (i.e., a sign of relaxation) demonstrating the physiological effects of Shinrin-yoku (taking in the atmosphere of the forest) (Park et al., 2007; Tsunetsugu et al., 2010). Forest bathing is also associated with short-term beneficial effects on stress-related issues and mild mood disorders (Antonelli et al., 2021), specifically in terms of technostress and study/work related symptoms of psychophysical stress. A campus forest walking program significantly increased health-promoting behaviors and parasympathetic nerve activity and decreased depression (Bang et al., 2017). Walking and physical activity are further associated with positive mood and increased self-esteem (Barton and Pretty, 2010).

Sometimes, sitting in a greenspace is enough to improve one’s mood. Ibes and Forestell (2020) demonstrate that sitting for 20 min in a greenspace located in central campus reduced participants’ mood disturbance relative to those who sat indoors. Also, practicing meditation in nature improves students’ mental health (Holt et al., 2019). Both direct and indirect contact with nature in campus settings increase energy, self-confidence and feelings of awe that lead to higher QoL, better overall mood, and lower perceived stress (Holt et al., 2019).

In this section, we have pointed to the kinds of processes that afford connection to one’s self, such as active and passive pursuits in nature. Open and green spaces as well as flexible, loose spaces allow a time-out from stress and provide opportunities for self-expression, and self-regulation, the psychological processes needed for a greater sense of connectedness.

## Concluding Comments

Biophilia is rooted in an inherent need for connectedness, and is activated through *affordances for connectedness* expressed through a pattern language for campus settings. The connectedness processes (outlined in our diagram) demonstrate how green/open spaces influence students’ QoL in its different dimensions, and contribute to fill the missing link between design and policies for the promotion of green university campuses. Connectedness is used as a guiding relational concept (Chan et al., 2018) to link everyday campus experience to students’ QoL.

Our conceptual framework aligns with previous efforts to show how students can connect to nature in campus settings by activating three levels of biophilic integration: indirect, incidental, and intentional (Abdelaal, 2019) and how such engagements result in restorative experiences (Gulwadi et al., 2019). Design elements and open space assessments in campus settings are often discussed broadly without clear links to the concept of connectedness (Lau et al., 2014). Our framework shows connectedness can be promoted in campus settings via direct, indirect, and place-based experiences of nature. Current university agendas treat the student as a consumer but a more relational view can account for core dimensions of human experience such as connectedness (Baumeister and Leary, 1995). Connection with campus nature is critically important to human health and well-being, especially in periods of lockdown, such as the COVID-19 pandemic that led to great isolation and separated students from their established social networks.

University campuses can also engage in environmental sustainability and innovation (Beynaghi et al., 2016) in addition to promoting students’ health and QoL. The diverse meanings of a campus, can be promoted through place-based education. In line with biophilic principles, place-based learning fosters connections with the local environment transforming placeless academic education from abstracted knowledge to learning ‘as a way of connecting’ (to environments, others, and self). We envision campus settings as niches for connectedness through the activation of biophilic potentialities and campus affordances. The proposed framework in this study articulates QoL in terms of enhanced connectedness to pose the following questions: What kinds of nature would be desirable on a campus setting with student-specific QoL in mind? What are the practical insights to enhance students’ connectedness?

This framework contributes to further nature-based campus interventions. Interventions should focus both on changing the environment and on designing social systems that lead to greater connectedness. The design of social systems is crucial to accommodate a heterogeneous system of open spaces and to foster civic stewardship (especially during times of crises). Our framework highlights three connectedness dimensions and points to design patterns that support this basic affiliative motive. Future biophilic-inspired research and design should thus take these three dimensions of connectedness into account as they are evolutionarily functional and thus, promote students’ QoL, health, and well-being.

## Author Contributions

SA contributed to the conception and first draft of the manuscript. GB and PN wrote sections of the manuscript. All authors contributed to manuscript revision, read, and approved the submitted version.

## Conflict of Interest

The authors declare that the research was conducted in the absence of any commercial or financial relationships that could be construed as a potential conflict of interest.

## Publisher’s Note

All claims expressed in this article are solely those of the authors and do not necessarily represent those of their affiliated organizations, or those of the publisher, the editors and the reviewers. Any product that may be evaluated in this article, or claim that may be made by its manufacturer, is not guaranteed or endorsed by the publisher.
